# Black and Latinx Community Perspectives on COVID-19 Mitigation Behaviors, Testing, and Vaccines

**DOI:** 10.1001/jamanetworkopen.2021.17074

**Published:** 2021-07-15

**Authors:** Manuel E. Jimenez, Zorimar Rivera-Núñez, Benjamin F. Crabtree, Diane Hill, Maria B. Pellerano, Donita Devance, Myneka Macenat, Daniel Lima, Emmanuel Martinez Alcaraz, Jeanne M. Ferrante, Emily S. Barrett, Martin J. Blaser, Reynold A. Panettieri, Shawna V. Hudson

**Affiliations:** 1 Department of Pediatrics, Rutgers Robert Wood Johnson Medical School, New Brunswick, New Jersey; 2 Department of Family Medicine and Community Health, Rutgers Robert Wood Johnson Medical School, New Brunswick, New Jersey; 3 Department of Biostatistics and Epidemiology, Rutgers School of Public Health, Rutgers Environmental and Occupational Health Sciences Institute, Piscataway, New Jersey; 4 Rutgers School of Public Affairs and Administration, University-Community Partnerships, Newark, New Jersey; 5 Rutgers, the State University of New Jersey, University-Community Partnerships, Newark; 6 Department of Medicine, Rutgers Robert Wood Johnson Medical School, Rutgers Center for Advance Biotechnology and Medicine, New Brunswick, New Jersey; 7 Department of and Pathology, Rutgers Robert Wood Johnson Medical School, Rutgers Center for Advance Biotechnology and Medicine, New Brunswick, New Jersey; 8 Rutgers Institute for Translational Medicine and Science, Rutgers Robert Wood Johnson Medical School, New Brunswick, New Jersey

## Abstract

**Importance:**

Black and Latinx communities have been disproportionately affected by the COVID-19 pandemic, yet little work has sought to understand their perspectives.

**Objective:**

To explore the experiences of Black and Latinx communities during the pandemic to better understand their perspectives on COVID-19 mitigation behaviors (eg, mask wearing), testing, and vaccines.

**Design, Setting, and Participants:**

In this community-engaged qualitative study conducted with 18 community-based organizations and 4 health care organizations between November 19, 2020, and February 5, 2021, in New Jersey counties severely affected by the pandemic, group and individual interviews were used to purposively sample 111 Black and Latinx individuals. A total of 13 group interviews were organized by race/ethnicity and language: 4 English-speaking groups with Black participants (n = 34), 3 Spanish-speaking groups with Latinx participants (n = 24), and 4 English-speaking groups with Black and Latinx participants (n = 36). To understand the views of health care workers from these communities, 2 additional groups (n = 9) were convened and supplemented with individual interviews.

**Main Outcomes and Measures:**

Description of Black and Latinx participants’ experiences during the COVID-19 pandemic and their perspectives on mitigation behaviors, testing, and vaccines.

**Results:**

The study included 111 participants (87 women [78.4%]; median age, 43 years [range, 18-93 years]). Participants described the devastating effects of the pandemic on themselves, loved ones, and their community. Their experiences were marked by fear, illness, loss, and separation. These experiences motivated intense information seeking, mitigation behaviors, and testing. Nevertheless, vaccine skepticism was high across all groups. Participants did not trust the vaccine development process and wanted clearer information. Black participants expressed that they did not want to be subjects of experiments.

**Conclusions and Relevance:**

The remaining unknowns about new vaccines need to be acknowledged and described for Black and Latinx communities to make informed decisions. Ultimately, scientists and public officials need to work transparently to address unanswered questions and work collaboratively with trusted community leaders and health professionals to foster partnered approaches, rather than focusing on marketing campaigns, to eliminate vaccine skepticism.

## Key Points

**Question:**

What are the experiences of Black and Latinx communities during the COVID-19 pandemic, and how are these experiences associated with their perspectives on COVID-19 mitigation behaviors, testing, and vaccines?

**Findings:**

This community-engaged qualitative study found that fear, illness, and loss experienced during the pandemic motivated information seeking and mitigation behaviors; vaccine skepticism was high, as was demand for clearer information. Among Black participants, racism and medical experimentation were associated with distrust.

**Meaning:**

These findings suggest that perspectives on COVID-19 mitigation behaviors, testing, and vaccines among Black and Latinx communities are informed by devastating experiences, and transparent information from public officials is needed to eliminate vaccine skepticism.

## Teaser

This qualitative study explores the experiences of Black and Latinx communities during the pandemic to better understand their perspectives on COVID-19 mitigation behaviors (eg, mask wearing), testing, and vaccines.

## Introduction

As of May 2021, the US has the highest number of cases and deaths in the world. Within the US, the pandemic is disproportionately affecting Black and Latinx groups.^1,2^ For example, age-adjusted mortality rates for Black and Latinx Americans far exceed those for White Americans.^3^ Multiple factors are associated with this inequality, including comorbid conditions that increase susceptibility and disease severity.^4,5^ Disparities in COVID-19 outcomes are also a function of structural and institutional racism.^6^ Factors such as residential segregation, wealth inequality, and mass incarceration impact the ability of members from different racial/ethnic and socioeconomic groups to avoid infection and seek care.^7,8,9,10,11^ These factors are the legacy of slavery, Jim Crow laws (state and local laws enacted in the late 19th and early 20th centuries that enforced racial segregation in the southern United States), and discriminatory public health interventions that together are associated with a pervasive sense of distrust of public health authorities.^12,13^

Black and Latinx groups have been the target of multiple discriminatory health interventions.^14^ Experiences including experimentation during slavery, the Tuskegee Syphilis Study, and the contraception trials in Puerto Rican women, predispose Black and Latinx communities to skepticism about public health interventions.^12,15^ Today, this history provides critical context for the strategies needed to fight the COVID-19 pandemic, including Centers for Disease Control and Prevention–recommended mitigation behaviors (eg, mask wearing, handwashing, and physical distancing), testing, and vaccines.^16^ In recent surveys, Black, Latinx, and low-income respondents were much less likely to report trust in public health officials in association with COVID-19, compared with White respondents.^17^ Black and Latinx adults are also more likely to “wait and see” before receiving the COVID-19 vaccination.^18^

Surveys, news reports, and anecdotes have brought attention to the inequities experienced by Black and Latinx communities during the COVID-19 pandemic.^17,19^ To date, little work has delved deeply into the experiences of these communities to better understand their perspectives on COVID-19 mitigation behaviors, testing, and vaccines. Such information is critical to develop appropriate public health messages and strategies. Therefore, this study explores the experiences of Black and Latinx adults during the pandemic to understand how these experiences are associated with their perspectives on COVID-19 public health strategies.

## Methods

We conducted online group and individual interviews as part of NJ HEROES TOO (New Jersey Healthcare Essential Worker Outreach and Education Study–Testing Overlooked Occupations),^20,21^ funded by the National Institutes of Health Rapid Acceleration of Diagnostics–Underserved Populations (RADx-UP) Initiative.^22^ The RADx-UP Initiative seeks to better understand disparities among underserved populations, including access to testing. We purposively sampled Black and Latinx individuals from New Jersey counties (ie, Essex, Middlesex, Passaic, and Union) with high rates of COVID-19 infections and deaths during the initial surge in 2020, high levels of poverty, and large concentrations of Black and Latinx populations. Adults older than 18 years of age who identified as Black or Latinx with English or Spanish as their primary language were eligible. We partnered with 18 community-based organizations and 4 health care organizations in these counties. Through biweekly online meetings, representatives helped with development of the research protocol, recruitment, and debriefing sessions to help interpret findings.^23^ This study was approved by the Rutgers Biomedical Health Sciences institutional review board and follows the Standards for Reporting Qualitative Research (SRQR) reporting guideline.^24^ All participants provided verbal consent prior to participation.

### Data Collection

We organized 13 group interviews and 8 individual interviews between November 19, 2020, and February 5, 2021, using a secure Zoom platform. Group interviews had a primary and secondary facilitator (M.E.J., D.H., D.L., and/or S.V.H.) with 2 study team members (Z.R.-N., M.B.P., M.M., and/or E.M.A.) for notetaking and technical assistance. Facilitators followed the interview guide, which we developed through literature review, the team’s experience, and partner feedback (eMethods in the Supplement). We adapted and added questions to explore emerging themes. Group interviews were organized by race/ethnicity and language: 4 English-speaking groups with Black participants, 3 Spanish-speaking groups with Latinx participants, and 4 English-speaking groups that included Black and Latinx participants (Figure). We also conducted 2 group interviews with Black and Latinx participants who worked in health care settings as ancillary or support staff, given their unique perspective as health care workers and community members. We supplemented health care worker group interviews with 8 individual interviews to accommodate their schedules. Group interviews lasted approximately 90 minutes, and individual interviews lasted 20 to 30 minutes. All interviews were recorded and transcribed. We used ATLAS.ti 8 software (ATLAS.ti) to facilitate data management.

**Figure.**
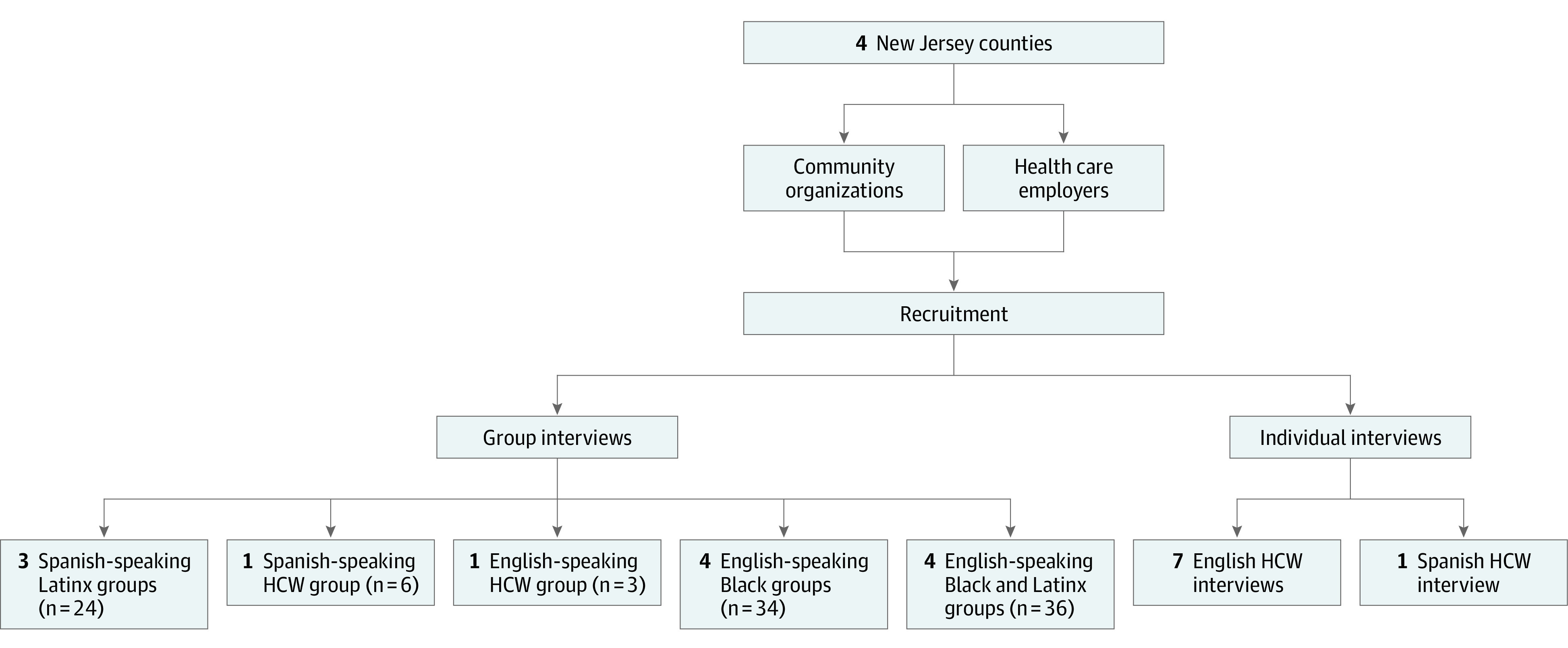
Organization of the NJ HEROES TOO (New Jersey Healthcare Essential Worker Outreach and Education Study–Testing Overlooked Occupations) to Obtain Perspectives on Community Concerns About COVID-19 Mitigation Behaviors, Testing, and Vaccination The study was conducted as part of the RADx-UP (Rapid Acceleration of Diagnostics–Underserved Populations) Initiative from November 19, 2020, through February 5, 2021, and included group and individual interviews of 111 individuals. HCW indicates health care worker.

### Data Analysis

We used an “editing” approach to analyze the data.^25^ Transcripts were first read openly to gain an initial sense of the group conversation. On a second reading, meaningful segments of text were highlighted, then cut, pasted, and rearranged to create 4- to 5-page summaries organized around emerging themes. We then analyzed the summaries together of groups with only Black participants to identify themes that were common to those groups; subsequently, we identified themes in summaries of groups with only Latinx participants in the same manner. Looking across both sets, we then identified the themes common to both groups. At that point, we analyzed the groups with Black and Latinx participants to confirm or disconfirm the existing findings and found that we had achieved data saturation—no new themes emerged from this analysis. Finally, we analyzed health care worker data, using the same process, which yielded similar findings; therefore, we include health care worker perspectives under our key themes.

## Results

We recruited 200 individuals, 64 of whom were not eligible and 25 did not attend, for a final sample of 111 individuals (87 women [78.4%]; 68 Black participants [61.3%] and 43 Latinx participants [38.7%]; median age, 43 years [range, 18-93 years]). The Table summarizes sample demographic characteristics, and the eTable in the Supplement summarizes demographic characteristics by group. We identified 2 key themes from the data: (1) the devastating effects of the pandemic motivated intense information seeking and mitigation behaviors and testing and (2) even within that context, vaccine skepticism was high (Box).

**Table.** Demographic Characteristics of Study Participants

Characteristic	Participants, No. (%) (N = 111)
Age, median (range), y	43 (18-93)
Sex	
Female	87 (78.4)
Male	24 (21.6)
Race/ethnicity	
Black or African American	68 (61.3)
Hispanic or Latinx	
White	3 (2.7)
Other	40 (36.0)
Educational level	
Doctoral or professional degree	7 (6.3)
Master’s degree	13 (11.7)
4-Year degree	33 (29.7)
Associate’s degree	8 (7.2)
Some college, no degree	21 (18.9)
High school diploma or equivalent	22 (19.8)
<High school	6 (5.4)
Household income, $	
≥150 000	6 (5.4)
100 000-149 999	15 (13.5)
75 000-99 999	7 (6.3)
50 000-74 999	16 (14.4)
25 000-49 999	24 (21.6)
<25 000	16 (14.4)
Refused or do not know	27 (24.3)
Total household members, No.	
1	1 (0.9)
2	24 (21.6)
3	20 (18.0)
4	22 (19.8)
5	22 (19.8)
≥6	19 (17.1)
Refused or missing	3 (2.7)
Health care worker	
No	88 (79.3)
Yes	23 (20.7)

Box. **Themes and Representative Quotations**

**Theme and Quotations**

**Devastating effects of the pandemic and intense information seeking, mitigation behaviors, and testing**

Devastation: illness, loss, separation, economic consequences, and fear
  “And then not only that, the hospital I was at, my son worked there, so it wasn’t like he can come by and walk into the room. He just knew where I was, but they wouldn’t let him in because of what was going on. So it was just kind of traumatic to him.”
  “…my husband stopped working, I couldn’t work because I had surgery a month ago; he stopped working for 2 months because there were a lot of infected people at his job and they had to close down.”
Information seeking, mitigation behaviors, and testing
  “I understand the purpose for it, but it does get a little uncomfortable at times, but you know you have to wear it just so I can protect myself and protect others.”
  “And I think just like everybody, I just want to make sure that if I’m symptomatic because we have had individuals that have tested and then they’re asymptomatic that I do not—I come to work, so I don’t want to have anyone become sick just because of me, right? So I want to make sure that I get tested often.”
Logistical problems with testing
  “In my husband’s experience, he gets [tested] regularly because he leaves the house. He had a test done with his saliva, he had to wait, not with a car, he had to walk; he had to wait 2 hours, without a car, there were many people. He was very afraid because people who go there are supposed to have doubts about whether they are infected or not; it was a lot of people; he didn’t think it was good that everyone was close.”
  “But there are a lot of people I’ve talked with who don’t have that access or who’ve had to pay privately to get tested. And these tests cost from $100 to $200 in some places and they’re not places that are nearby for people who—like, in the community of New Brunswick, the majority of people walk. Places have been extremely far; 30-40 minutes away.”

**Vaccine skepticism**

Serious questions
  “Everyone should know how this came to be, why it’s so sudden. I feel like right now, we’re seeing a lot of articles and we’re like okay. I guess it’s coming, but it’s still again unclear. We’re not really sure. So I feel like next year, as it’s supposed to become more prevalent, the government really needs to step up and start talking about okay. This is what’s going on. This is how it came to be, and not just telling you okay. Take it. It’s an option. If you want to live your life again, do it. I would like to know more. So that’s my opinion.”
  “…my concern as far as the vaccine, because I’ve been going on the FDA [US Food and Drug Administration] and following the safety surveillance of COVID-19. And I’m just like really interested in the adverse outcomes and the changes. Because as we know, like with vaccines, they say they’re helpful. Well, we’re going to talk about COVID right now. And I understand we have a lot of people dying. I have people close to me die, someone very dear. But at the same time, we’re all different far as it’s going to affect us all differently. So with these adverse outcomes, it may… we may react to it differently. So I’m really concerned about that.”
Distrust among Black participants
  “I’m not comfortable with this vaccine whatsoever…. [T]hey’re talking about lower-income communities getting the vaccine, the first minorities and Black and Latino communities. They want us to get it first, and I’m like no. That just doesn’t seem right…”
  “For me, honestly, I don’t trust the vaccine 100 percent… I have a lot of question marks around it. I wonder, how will it—if I take it, how will it affect me? I was watching the news the other day—I believe it was CNN—and they had gave this vaccine to a woman I think in another country. I can’t recall exactly where, but they were saying that she may have some fertility issues. That’s one of the side effects… I’m thinking about my children, how will it affect them… So how is that going to affect me as a Black woman with my Black sons?”

### Motivation for Information Seeking and Precautions

Participants shared stories filled with illness, loss, separation, economic consequences, and fear. In this context, mitigation behaviors and testing were seen as a means toward self-preservation and saving loved ones. Logistical challenges around testing were prominent among Latinx participants.

#### Illness, Loss, Separation, Economic Consequences, and Fear

Participants shared their experiences with loss and illness during the pandemic. As 1 participant shared, “The first impact certainly has been family. I lost my wife… When my wife died, I had it and my son had it.” Participants grieved for family members and friends. As noted by another participant, “a lot of my family and friends have expired, which has a large impact.” The pandemic separated family and friends as well as supportive social groups and communities (eg, churches) during these times of crisis, illness, and death. As participants were losing loved ones, restrictions on funeral services were “disheartening”: “My grandmother actually passed away, and she lived in [country]. And so, me and a group of our family members actually took a road trip and we were actually turned away by the borders because of everything that was going on. And it was a bit disheartening knowing that we weren’t able to see her off.”

Stories about the steep economic consequences of COVID-19 were common. Participants struggled to keep up with expenses and bills. The pandemic amplified existing difficulties: “I work here and there… Bills are piling up trying to figure out a way how to pay this. It’s just—pretty much it’s just at that everyday struggle just got even more harder.”

Some participants did not feel safe inside or outside their homes, and described uncertainty about who among them had the virus. Crowded living conditions resulted in contact with neighbors and housemates who had COVID-19: “In my house there are several people, and we are a little group. When we had a COVID case, we had to wear masks, we had to have the windows open, even though it was cold.”

Leaving home also resulted in high-risk exposures. Participants described the risks associated with transportation for necessities such as health care visits and food shopping: “Now I prefer to walk because once I took a cab with my son and the man picked up someone else, that person coughed beside me, the man didn’t wear a face mask, and from there I didn’t call for a cab again. I try to walk so I don't have to go through that; my son got a fever 3 days later and he went to take the test, thank God it was negative. But we got scared, he was worried about if we had caught it in that cab.”

#### Work-Related Fears

Participants described the risks they encountered at work and the implications of exposing loved ones: “And I took the job knowing that risk and I go there every weekend worried that I will get it… and that if I do, I’ll bring it back home to my family. It’s pretty scary.”

#### Information Seeking, Mitigation Behaviors, and Testing

Participants shared stories about staying informed about the pandemic and described relying on federal, state, and local resources, as well as traditional and social media. Some older participants expressed skepticism of the latter. Participants characterized mitigation behaviors and regular testing as their responsibility: “So you really, really, really have to take this serious. They say ‘wear that mask.’ If that’s all that’s going to help then you have to do [it], you should be concerned and follow that order.”

Some credited their mitigation behaviors with keeping them safe: “I don’t go out, I avoid contact with other people. If I have contact with other people, I take something to wash my hands… If I buy food, I put on rubbing alcohol… I’m a little panicked, but I think it’s the best thing I’ve done all this time because thankfully, none of us have gotten it.”

Although exchanges around mask wearing and regular testing were generally positive, multiple participants cited the inconvenience of mask wearing and the discomfort they experienced during nasopharyngeal sampling for testing. However, they felt that compliance was important, despite inconvenience, to protect their loved ones and others: “So it’s something where you know you’re not just testing for yourself. If you have loved ones at home, you’re testing for them. If you have elderly people at home with certain conditions, you’re testing for them, as well.”

#### Latinx Participants’ Logistical Problems With Testing

Some participants described logistical barriers with testing, such as finding test centers, long waits, exposure risks while waiting, and difficulty accessing results. Logistical barriers were especially prominent among Latinx participants owing to issues with testing site accessibility, transportation, and language barriers. Once participants located testing sites, they reported onerous experiences: “…they made me wait outside in the cold for 2 hours, in a line, and then, told us we’d be called in 4 or 5 hours… So, what are you going to do? It’s another city, far from your home. Are you going to go back home?”

These discussions highlighted the unique difficulties encountered by undocumented individuals, for whom different rules applied: “Yesterday I went to 2 different places … both of them were supposedly free, but you had to have a Social Security number. If you don’t, it’s not free. You have to pay $150… There isn’t much information for people who don’t have a Social Security number … I met a person who got it done… he had to go to New York because in Newark he didn’t find any… there isn’t the necessary information available for us to know how to go about this.”

### Vaccine Skepticism

Participants’ experiences during the pandemic did not translate into acceptance of the COVID-19 vaccine. All groups had serious questions and wanted clear information. Black participants, especially, discussed distrust of the vaccine.

#### Serious Questions About the Vaccine

Serious questions about vaccines emerged. Some participants questioned how a vaccine for a novel virus could be developed so rapidly, when other diseases have been around for decades without successful vaccines: “I’m not interested in [the vaccine]… It’s a lot of things that have been out here for hundreds of years that you couldn’t find a vaccine for, but this here, in less than a year, you come up with a vaccine that’s supposed to cure it. So how do you have the vaccine so fast?”

Participants expressed concerns that the vaccine development process including the clinical trials had been “rushed.” Participants questioned the extent to which scientists understood whether the vaccine would be safe. They worried about the short-term and long-term adverse effects: “I wouldn’t feel confident about the vaccine because I wouldn’t know what the long-term side effects would be.”

Participants wanted clear and transparent information on vaccine effectiveness. They questioned whether vaccines would work against COVID-19 variants. Participants also wanted information that was specific to their communities: “…I also think that doctors need to be well informed and transmit that information well to their patients… for example, our Hispanic community, which is different from the African or White communities—how has it affected us, what are our risks…”

Given their concerns, many participants wanted to see how others would respond first: “The only way I would get the vaccine would be if the same scientists who created it, and the executives and owners of the companies who created the vaccine, and the President and the entire Cabinet got the vaccine, then I would get the vaccine.”

#### Distrust Among Black Participants

Distrust of health care systems and government was cited by Black participants. The motives behind vaccination campaigns, especially those targeted toward Black and Latinx communities, were viewed with skepticism. Racism and the history of medical experimentation with the Black community were cited as reasons: “[I]f that comes out and we can prove that [the vaccine] is safe to take it, I will be there to take it, but I’m not going to be the first one… I have some suspicion always, growing up an African American male, we’ve been experimented on so often, and I don’t want to be a part of anybody’s experiment. But once I find out that it’s safe, for the good of humanity, I will participate at that point.”

## Discussion

The current study provides insight into the experiences of Black and Latinx communities during the COVID-19 pandemic and how these experiences are associated with their perspectives on mitigation behaviors, testing, and vaccines. Participants provided accounts of the devastation experienced in their communities that statistics cannot capture. Although these experiences motivated mitigation behaviors, members of these communities, who bore the brunt of the pandemic’s consequences, remained wary of vaccines because of questions about the development process, their effectiveness and safety, as well as distrust. Our findings should serve as a cautionary note to decision makers who would simply provide reassurance or dismiss wariness about the COVID-19 vaccine among communities of color as owing to lack of knowledge. Instead, we illustrate the need for clear, transparent information sharing and community-engaged strategies that can facilitate informed choices.

National surveys have found that Black and Latinx respondents are more likely to report engaging in COVID-19 mitigation behaviors than White respondents.^17,18,19,26^ Our findings provide insight into the disproportionate suffering motivating these behaviors. However, logistical barriers make testing difficult. Latinx participants, in particular, reported difficulty finding testing sites, transportation issues, and language barriers. Consistent with past work,^27^ this challenge was most pronounced for undocumented community members who were told to pay for testing, when they are ineligible for unemployment benefits and other assistance programs. Focused efforts to reduce logistical barriers and improve access to testing within underserved communities, regardless of documentation status, is imperative for these communities that are motivated to practice mitigating behaviors. Efforts to offer convenient testing options, accessible sites within walking distance, translated information, and transparency about free testing would help address these barriers.

Vaccine skepticism among Black and Latinx communities has been well documented.^28,29^ Although experiences during the pandemic motivated intensive information seeking and precautions, paradoxically, participants remained skeptical about a COVID-19 vaccine. Our findings add insight into the many unanswered questions that lead Black and Latinx individuals to take a “wait and see” approach to COVID-19 vaccination.^18^ Among Black participants, the experience of racism, past discriminatory interventions, and medical experimentation intensified their distrust. Our findings suggest that the success of vaccine campaigns among Black and Latinx communities will depend on transparent information and building on trusted relationships. Participants offered examples of the information they need to make a decision about vaccination, including information about how the vaccines were developed, effectiveness, adverse effects, and how others respond. It is imperative to be upfront about the risks of COVID-19 and what remains unknown about vaccines, because there is potential for backlash if vaccines are oversold.

Our findings illustrate how Black and Latinx communities are intensively seeking information, and skepticism can be intensified when information does not match experience. With the widespread availability of social media and alternative sources of information, the government needs to be transparent. Efforts that engage community leaders and trusted health professionals as partners who can engage in patient-centered processes that support informed choices about vaccination are needed. Incorporating primary care professionals and community organizations in vaccine distribution efforts would help facilitate engagement, while addressing structural barriers inherent in complicated online registration processes and navigating large systems (eg, retail chains) that disproportionately affect communities of color.

### Limitations

Our work has some limitations. First, while qualitative methods are well suited to understand the meaning and experiences of individuals, we are unable to establish prevalence of beliefs or behaviors. Second, we purposively sampled Black and Latinx participants from largely urbanized counties in one state; thus, our findings may not transfer to other racial/ethnic groups or rural settings. Third, the context of the COVID-19 pandemic is rapidly evolving and participants’ perspectives may change over time. Specifically, data collected in November 2020 before the vaccine rollout began may reflect stronger hesitation than data collected in February 2021. Fourth, this study focused on understanding community perspectives and therefore did not measure the extent to which perspectives translated into specific behaviors.

## Conclusions

This qualitative study offers insight into the experiences that motivate COVID-19 mitigation behaviors among Black and Latinx communities and why vaccine skepticism is high among these groups. Logistical barriers to testing must be addressed and vaccine skepticism needs to be taken seriously. Rather than investing in marketing campaigns to sell vaccines to reluctant consumers, transparent information, including what is yet unknown, is needed so that members of these communities can make informed decisions.

## Notes

Supplement. **eMethods.** NJ HEROES TOO – Interview Guide **eTable.** Sample Demographic Characteristics by Group
