# Supplementary material for: Black and Latinx Community Perspectives on COVID-19 Mitigation Behaviors, Testing, and Vaccines
Source: JAMA Netw Open. 2021 Jul 15;4(7):e2117074. doi: 10.1001/jamanetworkopen.2021.17074 (PMC8283554; doi:10.1001/jamanetworkopen.2021.17074)
Supplement: Supplement. — eMethods. NJ HEROES TOO – Interview Guide eTable. Sample Demographic Characteristics by Group [file jamanetwopen-e2117074-s001.pdf]

Supplementary Online Content

Jimenez ME, Rivera-Núñez Z, Crabtree BF, et al. Black and Latinx community perspectives on COVID-19 mitigation behaviors, testing, and vaccines. *JAMA Netw Open.* 2021;4(7):e2117074. doi:10.1001/jamanetworkopen.2021.17074

eMethods. NJ HEROES TOO – Interview Guide

eTable. Sample Demographic Characteristics by Group

This supplementary material has been provided by the authors to give readers additional information about their work.

© 2021 Jimenez ME et al. *JAMA Network Open.*

eMethods. NJ HEROES TOO – Interview Guide

Introductory Script

Welcome everyone. Thanks for taking the time to join us to talk about your views on testing for COVID-19. My name is [NAME] and I will lead today's discussion. Assisting me is [NAME] and we're in the [UNIT] at Rutgers University. The goal of today's conversation is for us to learn about how COVID-19 is impacting you and your community and your thoughts on testing, contact tracing and vaccines for COVID-19. Just to make sure we have a common understanding, by contact tracing we are referring to a method of reducing the spread of COVID-19 that involves identifying the people who have been in contact with a person infected with COVID-10 so they will not unknowingly spread the virus to others. We are having discussions like this with several groups across NJ and we are hoping to learn from you so that we can better design education materials about what we need to know about COVID-19 and testing. We thank you for sharing your time with us.

We want you to know there are no wrong answers or wrong ideas. We welcome differing points of view. Please feel free to share your thoughts and feelings even if they are different from what others have said. We are equally interested in all views.

Before we begin, I'd like to go over the points in the informed consent form you received. First, I want to highlight that we are recording this session through Zoom's audio/video recording function because we don't want to miss any of your comments. People often say very helpful things in these discussions, and we can't write fast enough to get them all down. We will be on a first name basis during this conversation, but we won't use your names in our reports so that what you share today will be private. The reports will be used to help develop effective ways to communicate with people in this community about COVID-19.

We began recording when we opened this Zoom meeting. The file of our recorded conversation will be uploaded through a secure web portal to a professional transcription company. The file will be temporarily stored on encrypted and password-protected Rutgers University server, and the files will be destroyed by a member of the research team after checking the transcript for accuracy.

The main risks/discomforts of participating in this study are the potential inconvenience of taking the time to take part in this conversation, and breach of confidentiality. All efforts, however, will be made to keep your personal information confidential, but total confidentiality cannot be guaranteed. Your name and other information that could identify you will be kept private. There will be a code number, so your actual name will not be used. Only the study researchers will be able to link the code number to your name. Your personal identifiers and research data will be stored in a secure area in the Robert Wood Johnson Medical School. Only the research team will have direct access. To help us protect your privacy, we have also obtained a Certificate of Confidentiality from the National Institutes of Health, which we can use to legally refuse to disclose information that may identify you if there was, for instance, a court subpoena. Where this Certificate wouldn't have an effect is if, for example, there was suspected child or elder abuse, some communicable diseases, or threats of harm to yourself or others.

There are no direct benefits from taking part in this study.

This meeting will last approximately 60-90 minutes depending on how talkative we all are. You can step away at any time if you need to do so. As a small way of saying thank you, we have a $30 gift card for each of you which we will send to you via email or regular mail depending on your preference.

Are there any questions before we begin?

OK, let's begin.

© 2021 Jimenez ME et al. *JAMA Network Open.*

GRAND TOUR QUESTIONS

1. Can you tell me about some of the ways in which COVID-19 has affected you?
    a. Related to your work role?
    b. Family?
    c. Community?

2. What are some of the things that people in your community go through when they have tested positive for COVID-19?
    - -To what extent does missed work impact people in your community?
    - -How do people in your community tend to treat people who have tested positive for COVID-19?
    - -To what extent have you seen judgmental reactions?
    - -What kind of information do you think would be helpful to you, your family, your community related to positive tests?

3. What are your feelings about COVID-19 contact tracing?
    - -How comfortable would you feel with being called by a contact tracer?
        - -For those who may have privacy concerns, please speak more about this.
        - -What would make you feel comfortable speaking with a contact tracer?
    - -What kind of information do you think would be helpful to you, your family, your community related to contact tracing?

4. Please share your thoughts about getting tested for COVID-19 (PROBE: How would you want to be approached for testing?).
    - -Under what circumstances would you seek testing for yourself? For your family?
    - -What kinds of concerns do you have related to getting tested?
    - -What would make you feel most safe to get tested for COVID-19?
    - -What kind of information do you think would be helpful to you, your family, your community related to testing for COVID-19?

5. What is your opinion about a possible future vaccine for COVID-19?
    - -What kinds of concerns, if any, do you have about COVID-19 vaccines?
    - -What information about COVID-19 vaccines would be helpful to you?
    - -What would make you feel most comfortable getting a COVID-19 vaccine?
    - -What kind of information do you think would be helpful to you, your family, your community related to COVID-19 vaccines?

6. We'd like to know where you get your information on COVID-19.
    - -Which sources of information do you trust most?
        - -Why?
    - -Which sources of information do you trust the least?
        - -Why?
    - -To what extent do the opinions of your favorite celebrities impact your views?
        - -Who are some admired celebrities in your community?

© 2021 Jimenez ME et al. *JAMA Network Open.*

7. Where do you think would be the most effective places to put educational material on COVID-19 in your community? Please be specific.

    -Which of the following venues do you feel would have the most impact in your community: radio, television, online news, online advertisements, billboards, bus signs.

© 2021 Jimenez ME et al. *JAMA Network Open.*

eTable. Sample Demographic Characteristics by Group

No.	Group	Age Median (Range)	Sex n (%)		Race/Ethnicity n (%)		Household Income n (%)		Total Household Members * n (%)		Education n (%)		Healthcare Worker n (%)	
1	Black (n=9)	71 (55-93)	Female	9 (100)	Black	9 (100)	$150,000+	1 (11.1)	6+	0 (0)	Professional/ Doctoral Degree	0 (0)	Yes	0 (0)
			Male	0 (0)	Latinx	0 (0)	$100,000 to 149,999	0 (0)	5	0 (0)	Master's Degree	2 (22.2)	No	9 (100)
							$75,000 to 99,000	2 (22.2)	4	2 (22.2)	4-Year Degree	2 (22.2)		
							$50,000 to 74,999	1 (11.1)	3	1 (11.1)	Associate Degree	0 (0)		
							$25,000 to 49,999	1 (11.1)	2	5 (55.6)	Some College, No Degree	5 (55.6)		
							< 25,000	1 (11.1)	1	0 (0)	High School Diploma	0 (0)		
							Refused	3 (33.3)	Missing	1 (11.1)	< High School	0 (0)		
											Missing	0 (0)		
2	Black (n=11)	48 (26-78)	Female	7 (63.6)	Black	11 (100)	$150,000+	0 (0)	6+	1 (9.1)	Professional/ Doctoral Degree	1 (9.1)	Yes	1 (9.1)
			Male	4 (36.3)	Latinx	0 (0)	$100,000 to 149,999	0 (0)	5	1 (9.1)	Master's Degree	1 (9.1)	No	10 (90.9)
							$75,000 to 99,000	2 (18.1)	4	3 (27.3)	4-Year Degree	3 (27.3)		
							$50,000 to 74,999	3 (27.3)	3	3 (27.3)	Associate Degree	2 (18.2)		
							$25,000 to 49,999	1 (9.1)	2	3 (27.3)	Some College, No Degree	2 (18.2)		
							< 25,000	1 (9.1)	1	0 (0)	High School Diploma	2 (18.2)		
							Refused	4 (36.4)	Missing	0 (0)	< High School	0 (0)		
											Missing	0 (0)		
3	Black (n=10)	48.5 (25-68)	Female	9 (90)	Black	10 (100)	$150,000+	1 (10.0)	6+	0 (0)	Professional/ Doctoral Degree	2 (20.0)	Yes	0 (0)
			Male	1 (10)	Latinx	0 (0)	$100,000 to 149,999	2 (20.0)	5	1 (10.0)	Master's Degree	2 (20.0)	No	10 (100)
							$75,000 to 99,000	1 (10.0)	4	3 (30.0)	4-Year Degree	5 (50.0)		
							$50,000 to 74,999	1 (10.0)	3	4 (40.0)	Associate Degree	0 (0)		
							$25,000 to 49,999	2 (20.0)	2	2 (20.0)	Some College, No Degree	1 (10.0)		
							< 25,000	2 (20.0)	1	0 (0)	High School Diploma	0 (0)		
							Refused	1 (10.0)	Missing	0 (0)	< High School	0 (0)		

											Missing	0 (0)		
**4**	**Black (n=4)**	44.5 (41-69)	Female	1 (25)	Black	4 (100)	$150,000+	0 (0)	6+	1 (25.0)	Professional/ Doctoral Degree	0 (0)	Yes	1 (25.0)
			Male	3 (75)	Latinx	0 (0)	$100,000 to 149,999	0 (0)	5	1 (25.0)	Master's Degree	0 (0)	No	3 (75.0)
							$75,000 to 99,000	0 (0)	4	1 (25.0)	4-Year Degree	0 (0)		
							$50,000 to 74,999	0 (0)	3	0 (0)	Associate Degree	1 (25.0)		
							$25,000 to 49,999	2 (50.0)	2	1 (25.0)	Some College, No Degree	1 (25.0)		
							< 25,000	0 (0)	1	0 (0)	High School Diploma	2 (50.0)		
							Refused	2 (50.0)	Missing	0 (0)	< High School	0 (0)		
											Missing	0 (0)		
**5**	**Latinx (n=8)**	40 (29-47)	Female	8 (100)	Black	0 (0)	$150,000+	0 (0)	6+	4 (50.0)	Professional/ Doctoral Degree	1 (12.5)	Yes	1 (12.5)
			Male	0 (0)	Latinx	8 (100)	$100,000 to 149,999	1 (12.5)	5	3 (37.5)	Master's Degree	0 (0)	No	7 (87.5)
							$75,000 to 99,000	0 (0)	4	0 (0)	4-Year Degree	1 (12.5)		
							$50,000 to 74,999	0 (0)	3	0 (0)	Associate Degree	1 (12.5)		
							$25,000 to 49,999	2 (25.0)	2	1 (12.5)	Some College, No Degree	0 (0)		
							< 25,000	3 (37.5)	1	0 (0)	High School Diploma	1 (12.5)		
							Refused	2 (25.0)	Missing	0 (0)	< High School	4 (50.0)		
											Missing	0 (0)		
**6**	**Latinx (n=6)**	42 (26-44)	Female	6 (100)	Black	0 (0)	$150,000+	0 (0)	6+	1 (16.7)	Professional/ Doctoral Degree	0 (0)	Yes	0 (0)
			Male	0 (0)	Latinx	6 (100)	$100,000 to 149,999	0 (0)	5	1 (16.7)	Master's Degree	0 (0)	No	6 (100)
							$75,000 to 99,000	0 (0)	4	1 (16.7)	4-Year Degree	1 (16.7)		
							$50,000 to 74,999	0 (0)	3	1 (16.7)	Associate Degree	0 (0)		
							$25,000 to 49,999	3 (50.0)	2	2 (33.3)	Some College, No Degree	1 (16.7)		
							< 25,000	1 (16.7)	1	0 (0)	High School Diploma	3 (50.0)		
							Refused	2 (33.3)	Missing	0 (0)	< High School	1 (16.7)		
											Missing	0 (0)		

© 2021 Jimenez ME et al. *JAMA Network Open.*

**7**	**Latinx (n=10)**	33 (18-57)	Female	8 (80.0)	Black	0 (0)	$150,000+	0 (0)	6+	1 (10.0)	Professional/ Doctoral Degree	0 (0)	Yes	0 (0)
			Male	2 (20.0)	Latinx	10(100)	$100,000 to 149,999	0 (0)	5	4 (40.0)	Master's Degree	0 (0)	No	10 (100)
							$75,000 to 99,000	0 (0)	4	2 (20.0)	4-Year Degree	3 (30.0)		
							$50,000 to 74,999	0 (0)	3	2 (20.0)	Associate Degree	0 (0)		
							$25,000 to 49,999	3 (30.0)	2	0 (0)	Some College, No Degree	3 (30.0)		
							< 25,000	2 (20.0)	1	1 (10.0)	High School Diploma	3 (30.0)		
							Refused	5 (50.0)	Missing	0 (0)	< High School	0 (0)		
											Missing	1(10.0)		
**8**	**Black & Latinx (n=9)**	31 (20-38)	Female	6 (67.7)	Black	6 (67.7)	$150,000+	1 (11.1)	6+	0 (0)	Professional/ Doctoral Degree	0 (0)	Yes	1 (11.1)
			Male	3 (33.3)	Latinx	3 (33.3)	$100,000 to 149,999	4 (44.4)	5	3 (33.3)	Master's Degree	2 (22.2)	No	8 (88.9)
							$75,000 to 99,000	0 (0)	4	3 (33.3)	4-Year Degree	4 (44.4)		
							$50,000 to 74,999	1 (11.1)	3	1 (11.1)	Associate Degree	0 (0)		
							$25,000 to 49,999	2 (22.2)	2	2 (22.2)	Some College, No Degree	2 (22.2)		
							< 25,000	0 (0)	1	0 (0)	High School Diploma	1 (11.1)		
							Refused	1 (11.1)	Missing	0 (0)	< High School	0 (0)		
											Missing	0 (0)		
**9**	**Black & Latinx (n=9)**	48 (26-78)	Female	8 (88.9)	Black	6 (67.7)	$150,000+	1 (11.1)	6+	1 (11.1)	Professional/ Doctoral Degree	0 (0)	Yes	1 (11.1)
			Male	1 (11.1)	Latinx	3 (33.3)	$100,000 to 149,999	2 (22.2)	5	2 (22.2)	Master's Degree	2 (22.2)	No	8 (88.9)
							$75,000 to 99,000	0 0)	4	1 (11.1)	4-Year Degree	4 (44.4)		
							$50,000 to 74,999	0 (0)	3	2 (22.2)	Associate Degree	1 (11.1)		
							$25,000 to 49,999	1 (11.1)	2	3 (33.3)	Some College, No Degree	2 (22.2)		
							< 25,000	2 (22.2)	1	0 (0)	High School Diploma	0 (0)		
							Refused	3 (33.3)	Missing	0 (0)	< High School	0 (0)		
											Missing	0 (0)		
**10**		38 (22-54)	Female	8 (80.0)	Black	7 (70.0)	$150,000+	1 (10.0)	6+	3 (30.0)	Professional/ Doctoral Degree	1 10.(0)	Yes	1 (10)

© 2021 Jimenez ME et al. *JAMA Network Open.*

	**Black & Latinx (n=10)**		Male	2 (20.0)	Latinx	3 (30.0)	$100,000 to 149,999	2 (20.0)	5	1 (10.0)	Master's Degree	1 (10.0)	No	9 (90)
							$75,000 to 99,000	1 10.0)	4	1 (10.0)	4-Year Degree	4 (40.0)		
							$50,000 to 74,999	1 (10.0)	3	5 (50.0)	Associate Degree	1 (10.0)		
							$25,000 to 49,999	3 (30.0)	2	0 (0)	Some College, No Degree	0 (0)		
							< 25,000	1 (10.0)	1	0 (0)	High School Diploma	3 (30.0)		
							Refused	1 (10.0)	Missing	0 (0)	< High School	0 (0)		
											Missing	0 (0)		
**11**	**Black & Latinx (n=8)**	49 (24-70)	Female	0 (0	Black	7 (87.5)	$150,000+	1 (12.5)	6+	3 (37.5)	Professional/ Doctoral Degree	1 (12.5)	Yes	0 (0)
			Male	8 (100)	Latinx	1 (12.5)	$100,000 to 149,999	2 (25.0)	5	1 (12.5)	Master's Degree	1 (12.5)	No	8 (100)
							$75,000 to 99,000	0 (0)	4	0 (0)	4-Year Degree	0 (0)		
							$50,000 to 74,999	1 (12.5)	3	1 (12.5)	Associate Degree	1 (12.5)		
							$25,000 to 49,999	1 (12.5)	2	1 (12.5)	Some College, No Degree	1 (12.5)		
							< 25,000	2 (25.0)	1	0 (0)	High School Diploma	4 (50.0)		
							Refused	1 (12.5)	Missing	2 (25.0)	< High School	0 (0)		
											Missing	0 (0)		
**12**	**HCW (n=3)**	43 (33-54)	Female	3 (100)	Black	3 (100)	$150,000+	0 (0)	6+	1 (33.3)	Professional/ Doctoral Degree	0 (0)	Yes	3 (100)
			Male	0 (0)	Latinx	0 (0)	$100,000 to 149,999	0 (0)	5	0 (0)	Master's Degree	0 (0)	No	0 (0)
							$75,000 to 99,000	0 (0)	4	2 (67.7)	4-Year Degree	1 (33.3)		
							$50,000 to 74,999	0 (0)	3	0 (0)	Associate Degree	0 (0)		
							$25,000 to 49,999	2 (67.7)	2	0 (0)	Some College, No Degree	1 (33.3)		
							< 25,000	0 (0)	1	0 (0)	High School Diploma	1 (33.3)		
							Refused	1 (33.3)	Missing	1 (11.1)	< High School	0 (0)		
											Missing	0 (0)		
**13**	**HCW (n=6)**	42 (25-49)	Female	6 (100)	Black	0 (0)	$150,000+	0 (0)	6+	1 (16.7)	Professional/ Doctoral Degree	1 (16.7)	Yes	6 (100)
			Male	0 (0)	Latinx	6 (100)	$100,000 to 149,999	1 (16.7)	5	4 (66.7)	Master's Degree	1 (16.7)	No	0 (0)
							$75,000 to 99,000	1 (16.7)	4	0 (0)	4-Year Degree	3 (33.7)		

© 2021 Jimenez ME et al. *JAMA Network Open.*

							$50,000 to 74,999	2 (33.3)	3	0 (0)	Associate Degree	0 (0)		
							$25,000 to 49,999	1 (16.7)	2	1 (16.7)	Some College, No Degree	0 (0)		
							< 25,000	0 (0)	1	0 (0)	High School Diploma	0 (0)		
							Refused	1 (16.7)	Missing	0 (0)	< High School	1 (16.7)		
											Missing	0 (0)		
**N/A**	**HCW (n=8)**	52 (38-58)	Female	8 (100)	Black	5 (62.5)	$150,000+	0 (0)	6+	2 (25.0)	Professional/ Doctoral Degree	0 (0)	Yes	8 (100)
			Male	0 (0)	Latinx	3 (37.5)	$100,000 to 149,999	1 (12.5)	5	0 (0)	Master's Degree	1 (12.5)	No	0 (0)
							$75,000 to 99,000	0 (0)	4	3 (37.5)	4-Year Degree	2 (25.0)		
							$50,000 to 74,999	4 (50.0)	3	0 (0)	Associate Degree	1 (12.5)		
							$25,000 to 49,999	2 (25.0)	2	3 (37.5)	Some College, No Degree	2 (25.0)		
							< 25,000	1 (12.5)	1	0 (0)	High School Diploma	2 (25.0)		
							Refused	0 (0)	Missing	0 (0)	< High School	0 (0)		
											Missing	0 (0)		

* Total household members includes participants

© 2021 Jimenez ME et al. *JAMA Network Open.*
